# Spermidine attenuates bleomycin-induced lung fibrosis by inducing autophagy and inhibiting endoplasmic reticulum stress (ERS)-induced cell death in mice

**DOI:** 10.1038/s12276-020-00545-z

**Published:** 2020-12-14

**Authors:** Ae Rin Baek, Jisu Hong, Ki Sung Song, An Soo Jang, Do Jin Kim, Su Sie Chin, Sung Woo Park

**Affiliations:** 1 Division of Allergy and Respiratory Medicine, Department of Internal Medicine, Soonchunhyang University Bucheon Hospital, 14584 Gyeonggi-Do, South Korea; 2 Department of Pathology, Soonchunhyang University Bucheon Hospital, 14584 Gyeonggi-Do, South Korea

**Keywords:** Immunology, Diseases

## Abstract

Spermidine is an endogenous biological polyamine that plays various longevity-extending roles and exerts antioxidative, antiaging, and cell growth-promoting effects. We previously reported that spermidine levels were significantly reduced in idiopathic pulmonary fibrosis (IPF) of the lung. The present study assessed the potential beneficial effects of spermidine on lung fibrosis and investigated the possible mechanism. Lung fibrosis was established in mice using bleomycin (BLM), and exogenous spermidine was administered daily by intraperitoneal injection (50 mg/kg in phosphate-buffered saline). BLM-induced alveolar epithelial cells showed significant increases in apoptosis and endoplasmic reticulum stress (ERS)-related mediators, and spermidine attenuated BLM-induced apoptosis and activation of the ERS-related pathway. Senescence-associated β-gal staining and decreased expression of p16 and p21 showed that spermidine ameliorated BLM-induced premature cellular senescence. In addition, spermidine enhanced beclin-1-dependent autophagy and autophagy modulators in IPF fibroblasts and BLM-induced mouse lungs, in which inflammation and collagen deposition were significantly decreased. This beneficial effect was related to the antiapoptotic downregulation of the ERS pathway, antisenescence effects, and autophagy activation. Our findings suggest that spermidine could be a therapeutic agent for IPF treatment.

## Lung fibrosis: Spermidine, anti-aging compound inhibit lung fibrosis in mice

A natural widely occurring molecule with anti-aging properties may offer a new therapeutic option for patients with idiopathic pulmonary fibrosis(IPF). Sung Woo Park and colleagues from Soonchunhyang University Bucheon Hospital, South Korea, administered the anti-aging compound spermidine to mice with bleomycin-induced lung fibrosis. The team had previously shown that IPF lung express lower levels of spermidine than do healthy individuals. In the current investigations, the researchers demonstrated that treatment of spermidine attenuate inflammation and reduce collagen deposition in mouse lungs. On a molecular level, spermidine limited oxidative stress induced cell death and growth arrest by preventing the build-up of misfolded proteins and by activating pathways for the removal of dysfunctional components from the cell.

## Introduction

Idiopathic pulmonary fibrosis (IPF) is a progressive, devastating lung disease characterized by alveolar epithelial cell (AEC) injury and the subsequent proliferation of activated fibroblasts known as myofibroblasts. The accumulation of myofibroblasts is responsible for the excessive deposition of extracellular matrix (ECM), resulting in irreversible distortion of the lung parenchymal structure^1,2^. Although the exact mechanisms of the development of lung fibrosis remain unclear, repeated injury and shortened survival of AECs have been recognized as initiating events^3,4^.

The precise mechanism of AEC damage remains unclear, but increased ERS or the accumulation of unfolded proteins in AECs have recently been implicated in IPF pathogenesis^5,6^. In addition, because the incidence of IPF increases with age, age-related mechanisms such as telomerase attrition and increased AEC senescence are also important drivers of the abnormal repair process during the initiation of fibrosis^7,8^.

Spermidine is a naturally occurring polyamine and is ubiquitous in living organisms as a polycation^9,10^. Polyamines are involved in various biological processes, including replication, transcription, translation, posttranslational modification, and membrane stability^11^, and they regulate cellular proliferation, differentiation, and apoptosis^12,13^. Previous studies have suggested that polyamines have multiple effects, including antioxidant and anti-inflammatory effects, in various animal disease models^14^. Eisenberg et al.^15^ reported that the exogenous administration of spermidine prolongs the life span of several organism models and significantly reduces age-related oxidative protein damage in mice. In addition, natural endogenous spermidine has prominent cardioprotective and neuroprotective effects and stimulates anticancer immunosurveillance in animal models^14^.

We recently reported that spermidine levels were decreased in the IPF lung compared to healthy controls^16^. Although the beneficial effects of spermidine have been reported in various chronic inflammatory diseases, its role in and effects on lung fibrosis have not yet been elucidated. Given the antiaging and antioxidative activities of spermidine, we hypothesized that spermidine may have beneficial effects on lung fibrosis, particularly in regulating cellular senescence and ERS-induced cell death.

Hence, in this study, we examined whether spermidine exerted beneficial effects on experimental lung fibrosis. The mechanism of the beneficial effects of spermidine was determined using AEC culture models. The therapeutic implication of spermidine in lung fibrosis development was evaluated in a mouse model of bleomycin (BLM)-induced lung fibrosis.

## Materials and methods

### Reagents and antibodies

The antibodies used were rabbit anti-p16 (#ab51243; Abcam, Cambridge, UK), rabbit anti-p-Rb (#8516; Cell Signaling Technology, Danvers, MA, USA), rabbit anti-CHOP (#5554; Cell Signaling Technology), rabbit anti-GRP78/Bip (#3177; Cell Signaling Technology), rabbit anti-ATF-6a (#sc22799; Santa Cruz Biotechnology, Santa Cruz, CA, USA), rabbit anti-IRE-1 (#ab37073; Abcam), rabbit anti-light chain (LC) 3B (#3868S; Cell Signaling Technology), rabbit anti-autophagy related gene 7 (ATG7) (#8558S; Cell Signaling Technology), rabbit anti-beclin-1 (#3738S; Cell Signaling Technology), rabbit anti-mTOR (#2972; Cell Signaling Technology), rabbit anti-mTOR (phospho) (#2971; Cell Signaling Technology), and mouse anti-β-actin (#A5316; Sigma-Aldrich, St. Louis, MO, USA). Spermidine (#S2626) and 4-PBA (#P21005) were purchased from Sigma-Aldrich. An LDH assay kit (#mk401; Takara) was used. The in situ cell death detection kit (#11684795910; Roche Diagnostics, Basel, Switzerland), cellular senescence (SA-β-gal staining) assay kit (#CBA-230; Cell Biolabs, San Diego, CA, USA), mouse interleukin-1 beta (IL-1β) enzyme-linked immunosorbent assay (ELISA) kit (#88701322; eBioscience, Inc., San Diego, CA, USA), mouse tumor necrosis factor*-*alpha (TNF-α) ELISA kit (#88732422; eBioscience, Inc.), and a mouse transforming growth factor-beta 1 (TGF-β1) ELISA kit (#DY1679; R&D Systems, Minneapolis, MN, USA) were also purchased.

### Mouse AEC isolation and culture

Primary mouse AECs were isolated from wild-type mice using a previously described protocol^17^ with minor modifications.

### Preparation of lung cell suspensions

Crude cell suspensions were prepared from C57BL/6 mice. The lungs were perfused with 0.9% NaCl using a 10-mL syringe fitted with a 21-gauge needle (BD Pharmingen, San Diego CA, USA) through the right ventricle of the heart until they were visually free of blood. A 21-gauge intravenous catheter was inserted into the trachea and secured tightly with a suture. The lungs were filled with 1–2 mL of dispase via the tracheal catheter and then allowed to collapse naturally, expelling part of the dispase. Low-melting-point agarose (l%, 0.45 mL, stored in a 45 °C water bath; Invitrogen, Paisley, UK) was slowly infused via the catheter. The lungs were immediately covered with crushed ice and incubated for 2 min. Then, they were removed and placed into 12 mL polypropylene culture tubes with 2 mL of dispase (Sigma-Aldrich), incubated for 45 min at room temperature, and kept on ice until the next step. The lungs were transferred into 7 mL of Dulbecco’s modified Eagle’s medium (DMEM) with 0.01% DNase I in 60 mm Petri dishes. The digested tissue was carefully separated from the airways with the curved edge of fine-tipped forceps and gently swirled for 5–10 min. The resulting suspension was successively filtered through 100 and 40 µm Falcon cell strainers and then through 25 µm nylon mesh. The filtered suspension was centrifuged at 130 × *g* for 8 min at 4 °C and resuspended in 10 mL of 10% fetal bovine serum (FBS) and 1% penicillin–streptomycin in 25 mM 4-(2-hydroxyethyl)-1-piperazineethanesulfonic acid (HEPES)-buffered DMEM.

### Magnetic purification of ATII cells from crude cell suspensions

The cells were incubated with biotinylated anti-CD32 (0.5 µg/million cells; BD Pharmingen, San Diego CA, USA) and biotinylated anti-CD45 (1.5 µg/million cells; BD Pharmingen) for 30 min at 37 °C. Meanwhile, streptavidin-coated magnetic particles (Thermo Fisher Scientific, Waltham, MA, USA) were washed twice in phosphate-buffered saline (PBS) (10 min each wash) in polypropylene culture tubes using a magnetic tube separator (Sigma-Aldrich). After incubation, the cells were centrifuged (130 × g for 8 min at 4 °C), resuspended in 7 mL of DMEM, added to the magnetic particles, and incubated with gentle rocking for 30 min at room temperature. At the end of the incubation, the tubes were attached to the magnetic tube separator with adhesive tape for 15 min. The cell suspension was aspirated from the bottom of the tube using a narrow-stemmed transfer pipette, centrifuged, and resuspended in a culture medium. Cell viability was determined by trypan blue staining. The purity of alveolar type II cells was assessed via pro-SPC immunofluorescence staining. As in previous studies, 4–8 × 10^5^ cells were isolated from a single mouse. In these samples, the purity of type II pneumocytes was 90–93%. The isolated cells were maintained in 10% FBS and 1% penicillin–streptomycin in 25 mM HEPES-buffered DMEM.

### Fibroblast isolation and culture

Primary human lung fibroblasts were obtained from SCH Biobank (Soonchunhyang Biobank, Bucheon, Korea) as previously described^18^. Briefly, the fibroblasts were derived from lung tissue obtained from IPF patients by video-assisted thoracoscopic biopsy. Lung samples were obtained by lung biopsy, usually 1 week after hospital admission. None of the patients had been treated with corticosteroids or immunosuppressive drugs at the time of the biopsy. Control fibroblasts were obtained from individuals who underwent a lobectomy to remove a primary lung tumor. No morphological evidence of disease was found in the tissue samples used for the isolation of control cells. Lung fibroblasts from IPF or control specimens were isolated by mechanical dispersal, and then trypsin digestion of tissues was used to mince them into 1 mm^2^ fragment. Fibroblast cultures were established in DMEM supplemented with 10% fetal calf serum, 100 U/mL penicillin, 100 mg/mL streptomycin, and 0.25 μg/mL amphotericin B. All cells were cultured at 37 °C in 95% air/5% CO_2_ until just prior to reaching confluence, which generally occurred in 1–2 weeks. After three passages, immunoblotting was performed using anti-vimentin antibodies on adherent cells harvested from the same culture vessels. All cells showed the morphological characteristics of fibroblasts. All experiments with IPF and control fibroblasts were performed on cells before the sixth passage.

### Apoptosis assay

Apoptotic cells in paraffin-embedded lung tissues were labeled using a terminal deoxynucleotidyl transferase (TdT)-mediated dUTP nick end labeling (TUNEL) assay kit (Roche Diagnostics, Basel, Switzerland). The numbers of TUNEL-positive (apoptotic) cells in three sections per sample were counted under a fluorescence microscope at ×400 magnification (Carl Zeiss Microsystems, Thornwood, NY, USA) as described previously^19^. Mouse AECs were exposed to vehicle or 10 µg/mL BLM with spermidine for 24 h after overnight serum starvation. The dose of spermidine administered was 100 µM. An annexin V-FITC/propidium iodide detection kit (BD Biosciences Pharmingen, San Diego, CA, USA) was used to determine the proportions of apoptotic and necrotic cells. Approximately, 1 × 10^6^ cells/mL were washed in PBS, surface stained, resuspended in binding buffer, incubated with FITC-conjugated annexin V and propidium iodide (PI) for 15 min in the dark at room temperature, washed, and resuspended in binding buffer.

### Immunoblotting

Protein were extracted from cells with lysis buffer (#78510; Thermo Fisher Scientific, Waltham, MA, USA) containing proteinase and phosphatase inhibitor cocktails (#05892970001 and #04906837001; Roche Diagnostics, Basel, Switzerland), and the samples were then centrifuged. Immunoblotting was performed as described previously. For each experiment, equal amounts of total protein were resolved by 10% sodium dodecyl sulfate-polyacrylamide gel electrophoresis. The proteins were transferred to a polyvinylidene difluoride membrane (#ISEQ00010; Millipore, Billerica, MA, USA) and incubated with a specific primary antibody for 2 h at 37 °C or for 24 h at 4 °C. After being washed several times with PBS containing Tween, the membrane was incubated with anti-rabbit immunoglobulin G (IgG) horseradish peroxidase (HRP)-linked secondary antibody (#7074; Cell Signaling Technology) or anti-mouse IgG HRP-linked secondary antibody (#7076; Cell Signaling Technology), followed by chemiluminescence detection (#34080; Thermo Fisher Scientific and #1705061; BIO-RAD, Berkeley, CA, USA) with a ChemiDoc^TM^ Touch Imaging System (BIO-RAD).

### SA-β-galactosidase staining

Senescent cells were analyzed using senescence-associated β-galactosidase (SA-β-gal) staining. Cells were grown in six-well plates, washed, fixed, and stained with the SA-β-gal cellular senescence assay kit (Cell Biolabs) according to the manufacturer’s protocol and as previously described^20^. The sections were examined under a microscope.

### Immunofluorescence staining of fibroblasts

Standard protocols for immunofluorescence microscopy were used as described previously^21^. Human primary fibroblasts were plated on cover-glass-bottom dishes and treated with or without with the indicated agents. The cells on the dishes were washed twice, fixed in 4% paraformaldehyde for 1 h, and washed three times after fixation. The cells were prepared and stained with the indicated primary antibodies (1:50) overnight at 4 °C. The slides were washed twice, incubated with chromogen-labeled secondary antibody (1:100) for 30 min, and washed three times after being stained. Images were obtained with a confocal microscope (Zeiss LSM 510 META). Autophagosomes were identified as LC3B-positive puncta, and autophagolysosomes were identified by the coexpression of LC3B and LAMP-1.

### Animal model of BLM-induced lung fibrosis and treatment with spermidine

Specific-pathogen-free C57BL/6 (Orient Bio Inc., Sungnam-Si, Gyeonggi-Do, Korea) mice were maintained under pathogen-free conditions. All animal procedures followed a protocol approved by the Institutional Animal Care and Use Committee of Soonchunhyang University Bucheon Hospital (SCHBC-animal-2016-011). On day 0, the mice were administered 3 U/kg BLM (Sigma-Aldrich) dissolved in a total volume of 200 µL endotoxin-free water by intratracheal instillation. To determine the proper therapeutic dose of spermidine, we performed a pilot study with a small number of mice. We treated bleomycin-induced mice with 10, 30, 50, and 100 mg/kg spermidine from day 10 to day 21, and then the analyzed inflammatory cells in BAL fluid. In addition, to evaluate the cellular toxicity of spermidine, LDH levels in BAL fluid were measured. We found that 50 mg/kg spermidine significantly diminished neutrophils and reduced LDH levels in the BAL fluid, indicating that 50 mg/kg spermidine was both effective and safe in mice (Supplementary Fig. 1). On days 10–21, the mice were administered 50 mg/kg spermidine (Sigma-Aldrich) dissolved in endotoxin-free water containing 0.3% dimethyl sulfoxide by intraperitoneal instillation. The sham control mice were treated with PBS only. On day 21, the mice were sacrificed with an overdose of a ketamine/xylazine mixture, and bronchoalveolar lavage (BAL) was performed by instilling 1 mL of PBS, which was gently retrieved, four times as described previously^22^. Cell numbers were measured using a hemocytometer, and differential cell counts were performed on slides prepared by cytocentrifugation and Diff-Quik staining (E. Merck KG, Darmstadt, Germany). The BAL fluid was centrifuged at 500 × *g* for 10 min, and the supernatant was stored at −70 °C. Some of the cell-free supernatants were used for biochemical analyses, such as lactate dehydrogenase (LDH) assays. The Institutional Animal Care and Use Committee of Soonchunhyang University Bucheon Hospital approved this study (SCHBC-2016-012).

### Histological assays

A portion of each left lung was fixed in 4% (v/v) buffered paraformaldehyde and embedded in paraffin. The tissue was cut into 5 µm-thick slices and stained with hematoxylin and eosin (H&E) or Masson’s trichrome. The right lung was snap-frozen by immersion in liquid nitrogen and stored at −80 °C prior to RNA and protein extraction. Lung sections were stained with H&E for histopathological analyses or with Masson’s trichrome to evaluate collagen content and distribution. The Ashcroft score for the evaluation of lung fibrosis has previously been described^23^.

### Immunohistochemical staining

The lung tissues were dehydrated and embedded in paraffin. For histological examination, 4 μm-thick sections on slides were treated with 1.4% H_2_O_2_-methanol for 30 min to block endogenous peroxidase. Then, nonspecific binding was blocked with 1.5% normal saline, and the slides were incubated with rabbit anti-p21 (1:200; #ab188224; Abcam, Cambridge, UK) and rabbit anti-p16 (1:100; #ab51243; Abcam) antibodies. The next day, the sections were incubated with ABC kit reagents (Vector Laboratories, Burlingame, CA, USA). The color reaction was developed by staining with a liquid DAB + substrate kit (Golden Bridge International Inc., Mukilteo, WA, USA). After immunohistochemical staining, the slides were counterstained with Harris’s hematoxylin for 1 min.

### Masson’s trichrome staining

The mouse lung sections were placed in Bouin’s solution at 56 °C for 1 h and then stained successively with Mayer’s hematoxylin solution for 5 min, Biebrich scarlet-acid fuchsin solution for 10 min, phosphomolybdic acid-phosphotungstic acid for 15 min, and aniline blue for 2 h (staining reagents from Sigma-Aldrich). The sections were examined under a microscope.

### Measurement of hydroxyproline in the mouse lung

To estimate the amount of collagen in the lung, the right lungs were used in a hydroxyproline assay (#MAK008; Sigma-Aldrich) according to the manufacturer’s protocol. Briefly, the lungs were weighed, homogenized in sterile water, and hydrolyzed in 12 N HCl at 120 °C for 3 h. The hydrolyzed samples were incubated with 4-(dimethylamino) benzaldehyde for 90 min at 60 °C, and the absorbance of oxidized hydroxyproline was measured at 560 nm. The amount of collagen is expressed in micrograms per milligram of lung tissue.

### Measurement of proinflammatory and profibrotic cytokine levels

ELISA kits were used to measure the concentrations of IL-1β, TNF-α, and the active form of TGF-β1 in lung tissue (IL-1β: #88701322; eBioscience, Inc., TNF-α: #88732422; eBioscience, Inc., TGF-β1: #DY1679; R&D Systems.) according to the manufacturers’ instructions.

### Statistical analyses

All data are expressed as the means ± standard error. The data were analyzed using the Kruskal–Wallis test, followed by the Mann–Whitney *U* test (with Bonferroni correction for intergroup comparisons), and *p* values < 0.05 were considered significant.

## Results

### Spermidine attenuates BLM-induced cell death in AECs

**Fig. 1:**
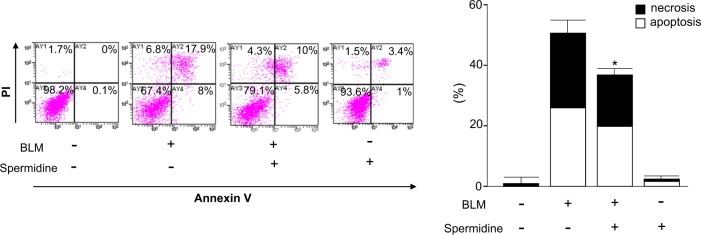
Antiapoptotic effects of spermidine on BLM-induced alveolar epithelial cells. Mouse primary AECs were exposed to BLM (10 µg/mL) and/or spermidine (100 µM) for 24 h in a serum-free medium. *p < 0.05 vs. apoptosis or necrosis in the BLM(+)/spermidine(−) group. Apoptosis was defined as both annexin V(+)/PI(+), and annexin V(+)/PI(−). Necrosis was defined as both PI(+)/annexin V(+) and PI(+)/annexin V(−). (*n* = 3).

BLM generates reactive oxygen species, which are responsible for the induction of cell death, and BLM is generally administered to establish lung fibrosis models for in vivo studies^24^. We examined the effects of spermidine on cell death in primary mouse AECs induced by BLM in an in vitro system. The selected concentrations of spermidine used in this experiment did not show any significant cytotoxicity at a concentration of 100 µM for up to 48 h of incubation, and cell viability remained >95% as measured using the MTT assay (data not shown). Twenty-four hours after BLM (10 µg/mL) administration in the presence of spermidine (100 µM) or vehicle, the proportions of apoptotic and necrotic cells were assessed by flow cytometry (Fig. 1). Spermidine significantly diminished BLM-induced increases in the fractions of apoptotic and necrotic cells (Fig. 1). These data indicated that spermidine significantly attenuated BLM-induced AEC apoptosis and necrosis.

### Spermidine inhibits BLM-induced senescence in AECs and mouse lungs

**Fig. 2:**
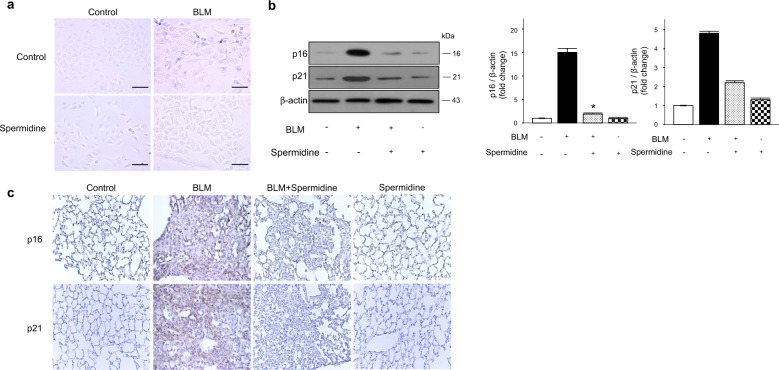
Spermidine inhibits senescence in BLM-induced mouse lung and alveolar epithelial cells. **a**. Mouse primary AECs were exposed to BLM (10 µg/mL) and/or spermidine (100 µM) for 24 h in serum-free medium. β-gal staining showed increased SA-β-gal activity in AECs after BLM exposure, and treatment with 100 µM spermidine reversed senescence in these cells. Scale bar, 50 μm. Mice were treated with BLM (3 U/kg) intratracheally on day 0, and spermidine (50 mg/kg/mouse/day) was administered intraperitoneally on days 10–21. Lung samples were collected on day 21. **b** Immunoblot analyses of the expression of p16 and p21 in whole lungs from mice. The intensity of each band was quantified by densitometry, and the data were normalized to β-actin. *p < 0.05 vs. the BLM(+)/spermidine(−)group. (*n* = 3) **c** Photographs of immunohistochemical staining showing the expression of p16 and p21 in mouse lungs. Original magnification ×100.

Cellular senescence is a conserved state of stable replicative arrest induced by aging-related stressors, including oxidative stress, DNA damage, and telomerase attrition^25^. Previous studies have shown that BLM, a fibrosis-inducing agent, induces cellular senescence^26^. Further evaluation by measuring SA-β-gal activity in cultured mouse AECs showed that BLM-induced SA-β-gal-positive staining was almost abolished by spermidine treatment (Fig. 2a). Next, we evaluated the effects of spermidine on BLM-induced senescence in mouse lung tissue. Immunoblot analyses showed that p16 and p21, which are molecular markers of senescence^27,28^, were increased in response to BLM administration compared to PBS treatment (Fig. 2b). Treatment with spermidine significantly downregulated the BLM-induced increases in p16 and p21 levels (Fig. 2b). Immunohistochemical analysis of mouse lung sections also showed that the BLM-induced increases in p16 and p21 were attenuated by spermidine (Fig. 2c). These results suggest that spermidine effectively inhibits the activation of senescence in BLM-induced AECs.

### Exogenous spermidine attenuates BLM-induced lung inflammation and fibrosis development

To clarify the therapeutic effects of spermidine on established BLM-induced lung fibrosis, we used an established BLM-induced lung fibrosis model as previously described with modifications^29^. Because excessive doses of spermidine are toxic to mammalian cells^10^, we treated BLM-induced mice with 10, 30, 50, and 100 mg/kg spermidine and measured BAL cell counts and LDH levels (Supplementary Fig. 1). BAL cell counts were measured, 50 mg/kg spermidine most dramatically decreased neutrophil numbers compared to those of the other groups (Supplementary Fig. 1a). LDH levels in BAL fluid were dose-dependently decreased by spermidine; however, 100 mg/kg spermidine increased LDH levels (Supplementary Fig. 1b). This result suggests that spermidine may cause cellular toxicity in certain doses in vivo. BLM was administered via the trachea on day 0, spermidine (50 mg/kg) was administered intraperitoneally on days 10–21, and lung samples were evaluated on day 21.

**Fig. 3:**
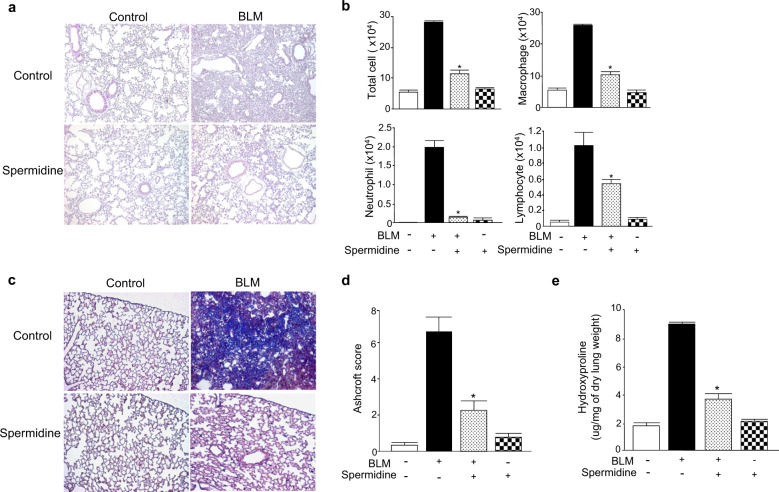
Spermidine attenuates BLM-induced lung inflammation and fibrosis in a mouse model. **a** Photographs of hematoxylin and eosin (H&E) staining of control and BLM-induced mouse lungs treated with spermidine (50 mg/kg/mouse/day) intraperitoneally on days 10–21. Lung samples were collected on day 21. Original magnification, ×100. **b** Cell counts in bronchoalveolar lavage (BAL) fluid collected on day 21. The total number of cells was counted using a hemocytometer. Differential cell counts in 500 cells isolated from BAL fluid were analyzed with Diff-Quik (*n* = 8/group). **c** Masson’s trichrome staining. Original magnification, ×200. **d** Quantification of lung fibrosis by the Ashcroft score (*n* = 8/group). The data are expressed as the mean ± standard error of the mean (SEM). **e** Collagen measurement by the hydroxyproline assay in control and BLM-induced mouse lungs with and without spermidine treatment (*n* = 8/group). *p < 0.05 vs. the BLM(+)/spermidine(−)group.

H&E staining showed that spermidine effectively reversed BLM-induced distortions in lung structure (Fig. 3a). Consistent with the H&E staining results, BAL fluid analyses showed that spermidine significantly attenuated lung inflammation and reduced the total numbers of macrophages, neutrophils, and lymphocytes (Fig. 3b). Furthermore, spermidine treatment clearly reduced lung fibrosis caused by collagen deposition, as indicated by Masson’s trichrome staining and Ashcroft scoring (Fig. 3c, d). The hydroxyproline assay further confirmed the antifibrotic effect of spermidine on BLM-induced fibrosis (Fig. 3e).

### Exogenous spermidine inhibits BLM-induced increases in profibrotic and proinflammatory mediators in mouse lungs

**Fig. 4:**
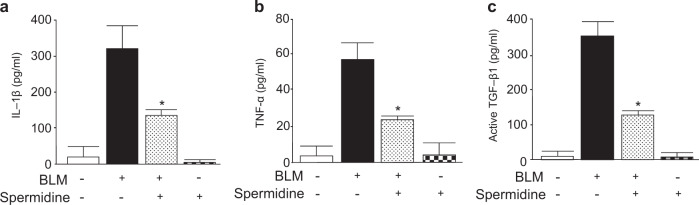
Spermidine inhibits BLM-induced increases in proinflammatory mediators and profibrotic cytokines in mouse lungs. **a** IL-1, **b** TNF-α, and **c** active TGF-β1 protein expression were measured using ELISA. The data are expressed as the mean ± SEM. *p < 0.05 vs. the BLM(+)/spermidine(−)group.

To determine whether spermidine inhibits the BLM-induced increases in proinflammatory cytokines and fibrotic mediators produced by macrophages and neutrophils, the protein levels of interleukin-1β (IL-1β), TNF-α, and active TGF-β1 in lung lysates were measured. All of the measured proinflammatory mediators and TGF-β1 in spermidine-treated mice were significantly decreased compared to those in BLM-induced mice (Fig. 4).

### Exogenous spermidine decreases the expression of ERS-related proteins in mouse lungs

**Fig. 5:**
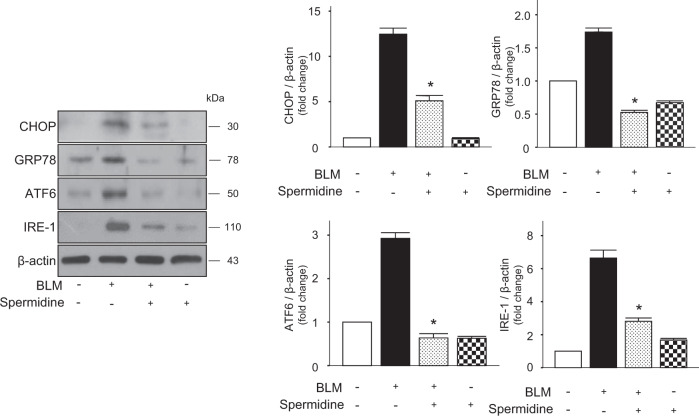
Spermidine decreases the expression of ERS-related proteins. The protein expression of CHOP, GRP78, ATF6, and IRE-1 was measured by immunoblotting. The intensity of each band was quantified by densitometry, and the data were normalized to β-actin. The data are expressed as the mean ± SEM. *p < 0.05 vs. the BLM(+)/spermidine(–)group. (*n* = 3).

Next, we investigated whether ERS was attenuated by spermidine-mediated protection against BLM-induced lung injury/fibrosis. ERS-related molecules in lung lysates were measured by immunoblotting. BLM-induced increases in ER-related proteins, including CHOP, GRP78, ATF6, and IRE-1, were also inhibited by spermidine (Fig. 5). This finding demonstrates that exogenous spermidine reduced BLM-induced lung injury/fibrosis and was associated with downregulation of the ERS pathway.

### Exogenous spermidine protects against BLM-induced apoptosis in mouse lungs

**Fig. 6:**
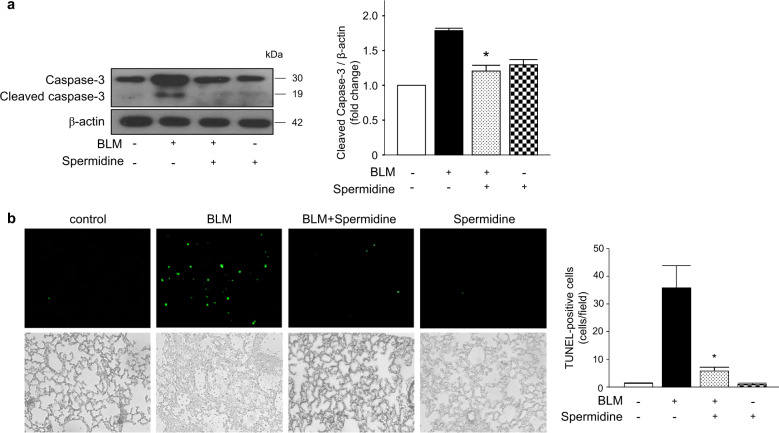
Spermidine attenuates BLM-induced apoptosis in the mouse lung. **a** Protein expression of the total and active forms of caspase-3 was measured by immunoblotting. The intensity of the active form of caspase-3 was quantified by densitometry, and the data were normalized to β-actin. The data are expressed as the mean ± SEM. **b** Tissues stained by the TUNEL method were observed at ×100 magnification, and the number of TUNEL-positive cells in a minimum of 20 fields per lung was counted (*n* = 8/group). *p < 0.05 vs. the BLM(+)/spermidine(−)group.

The ability to respond to perturbations in ER function is critical for cell survival, but chronic or unresolved ERS can lead to apoptosis^30^. First, we evaluated whether BLM-induced apoptotic cell death was mediated by excessive ERS. The ERS inhibitor 4-PBA was administered with BLM to mouse AECs, and apoptosis was measured by annexin-V/PI staining. As expected, the BLM-induced increases in apoptotic cell proportions were significantly decreased by 4-PBA (Supplementary Fig. 2). To determine whether spermidine inhibited BLM-induced apoptosis in vivo, we measured caspase-3 levels in mouse lungs. The protein levels of active caspase-3 were increased by BLM compared to those of the controls. The protein levels of the active form of caspase-3 in spermidine-treated mice were significantly decreased compared to those of BLM-induced mice (Fig. 6a). Similarly, a TUNEL assay revealed that approximately 75% of fewer apoptotic cells were present in the lungs of spermidine-treated mice than in the lungs of BLM-induced mice (Fig. 6b).

### Exogenous spermidine enhances autophagy in IPF fibroblasts and BLM-induced lungs

**Fig. 7:**
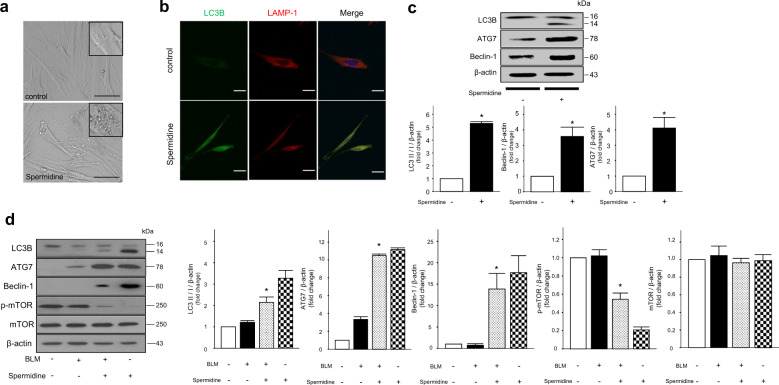
Spermidine enhances autophagy in idiopathic pulmonary fibrosis (IPF) fibroblasts and BLM-induced mouse lungs. IPF fibroblasts were treated with spermidine (100 μM) for 6 h. **a** Light microscopy images showing spermidine-induced vacuolar changes in fibroblasts; ×400, inset: ×1000. Scale bars, 50 μm. **b** Laser confocal microscopy was performed to measure LC3B and LAMP-1 by indirect immunofluorescence. Scale bars, 5 μm. **c** LC3B, ATG7, and beclin-1 expression was evaluated by immunoblotting. Densitometric analyses of independent experiments were performed. *p < 0.05 vs. the spermidine (−) group. The data are expressed as the mean ± SEM (*n* = 4). BLM-induced mice were treated with spermidine (50 mg/kg/mouse/day) intraperitoneally on days 10–21. Lung samples were collected on day 21. **d** The expression of autophagy-related proteins, including LC3 I/II, ATG7, beclin-1, mTOR, and p-mTOR, in lung lysates was measured by immunoblotting. Densitometric analyses of the experiments (*n* = 6/group). *p < 0.05 vs. the BLM(+)/spermidine(−)group.

Lung fibroblasts are the principal components of the interstitium and the main producers of the ECM, and the proliferation of (myo)fibroblasts is the main pathological finding in IPF^31^. Recent reports have implicated defective fibroblast autophagy in the pathogenesis of IPF^32,33^. We investigated whether spermidine regulated autophagic flux in IPF fibroblasts. First, spermidine (100 µM) led to the formation of cytoplasmic vacuoles after 6 h of treatment, suggesting the possible involvement of autophagy (Fig. 7a). The conversion of LC3-I to LC3-II is a hallmark of autophagy^34^. In addition, ATG7 and beclin-1 are key mediators of the canonical autophagy pathway^35^. We evaluated LC3B I/II expression in IPF fibroblasts by immunofluorescence and immunoblotting. Spermidine increased LC3B-expressing fibroblasts, as shown by immunofluorescence microscopy (Fig. 7b). Spermidine also increased the LC3B-I/II ratio and enhanced ATG7 and beclin-1 expression in fibroblasts (Fig. 7c). These data suggest that spermidine activates autophagy via a canonical pathway. In BLM-induced lungs, spermidine also enhanced the LC3 I/II ratio and the expression of ATG7 and beclin-1; however, p-mTOR expression decreased (Fig. 7d). These findings demonstrate that spermidine activates autophagy and that this effect may be beneficial in fibrotic lungs.

## Discussion

We showed that treatment with spermidine inhibited BLM-induced increases in AEC death, which is composed of the main pathophysiology of IPF. Our findings indicate that spermidine protects against BLM-induced lung inflammation and fibrosis. These beneficial effects are associated with suppressed upregulation of proinflammatory cytokines and neutrophilic inflammation and the prevention of apoptotic death in the lungs. In addition, we found other favorable effects of spermidine, particularly in the context of IPF pathogenesis, including the inhibition of cellular senescence, attenuation of ERS-mediated apoptosis, and activation of autophagy in primary lung fibroblasts and in vivo. This study is the first to report the therapeutic effects of spermidine on BLM-mediated experimental lung fibrosis and to elucidate the possible mechanism.

Senescence is a state of irreversible cell cycle arrest accompanied by an abnormal secretory profile and is thought to play a critical role in both development and wound repair^36^. As the incidence of IPF continuously increases with age, age-related mechanisms such as cellular senescence and telomere shortening have been proposed to be pathogenic drivers of lung fibrosis^36,37^. Recently, published reports have suggested that accelerated AEC senescence may contribute to the pathogenesis of IPF^38,39^. We showed that exogenous spermidine suppresses BLM-induced increases in cellular senescence in primary mouse AECs and in vivo (Fig. 2a). This result confirms that spermidine has antisenescence activity against oxidative stressors such as BLM.

The ER is an intracellular organelle that is responsible for the folding and sorting of proteins^40^. Various conditions, such as hypoxia, ischemia, and oxidative stress, can impair ER function and cause the accumulation of unfolded or misfolded proteins, resulting in ER stress, which leads to the activation of a signal response known as the unfolded protein response (UPR)^41^. According to previous reports, ER stress and UPR activation have been observed in the alveolar epithelium of patients with IPF^42,43^. Recently, ER stress and UPR have been linked to the development of lung fibrosis through the regulation of AEC apoptosis, epithelial–mesenchymal transition, and myofibroblast differentiation^44,45^. Previous studies have shown that spermidine acts as an inhibitor of ER stress in vitro and in vivo^46,47^. Our study indicates that spermidine also alleviates the BLM-induced increases in ATF6, IRE-1, GRP78, and CHOP in the lungs (Fig. 5). These results suggest that spermidine inhibits pulmonary ER stress and the activation of UPR signaling in BLM-induced lung fibrosis. Spermidine-mediated protection against BLM-induced lung fibrosis might be associated with the downregulation of ER stress in the lungs.

Polyamines have been implicated in apoptotic cell death in numerous studies in which cellular polyamines were altered either by overexpression or by the inhibition of biosynthetic enzymes^48^ and in studies with cells or animals with genetically modified polyamine pathways^49^. Either excessive accumulation or depletion of cellular polyamines is harmful to mammalian cells and can lead to cell death^48^. Polyamines may activate or inhibit apoptosis depending on the concentration and the specific apoptotic inducers. Regarding the possible mechanisms of the antiapoptotic effects, polyamine binding to DNA protects against DNA cleavage from ionizing radiation^50^; polyamines may also act as scavengers of reactive oxygen radicals^51^. However, excessive polyamines or the activation of amine oxidation can also cause oxidative stress and apoptosis by the generation of H_2_O_2_ and reactive aldehydes in cells^10,52^. In this study, 100 µM spermidine showed a beneficial effect by attenuating BLM-induced apoptosis in vitro (Fig. 1). We also found that although this concentration was not fatal, 100 mg/kg spermidine was toxic to BLM-induced mice after repeated administrations (Supplementary Fig. 1). These data suggest that the accumulation of excessive spermidine may be harmful both in vitro and in vivo.

When ER stress is elevated, cell apoptosis can be stimulated by the activation of caspase-12, which is then released into the cytoplasm, where it activates the final apoptotic pathway to sequentially activate caspase-9 and caspase-3. Our findings showed decreases in caspase-3 protein expression (Fig. 6a) and the number of TUNEL-positive cells suggests that spermidine inhibits BLM-induced apoptosis in the lung (Fig. 6b). To further determine whether ER stress was involved in BLM-induced apoptosis, we treated primary AECs with 4-PBA, a selective inhibitor of ERS, as previously described^53^. Treatment significantly reduced the proportion of apoptotic cells, which confirmed that the effects of spermidine occurred through ER-induced cell apoptosis (Supplementary Fig. 2).

Autophagy is a fundamental homeostatic process used by cells to degrade and recycle cellular proteins and remove damaged organelles. Autophagy is activated in response to intracellular or extracellular factors, such as hypoxia, ERS or oxidative stress, organelle damage, and pathogen infection^54^. Because spermidine is a physiological autophagy inducer^55^, we evaluated whether it reversed autophagy impairment in IPF fibroblasts and in vivo. Indeed, we found that spermidine enhanced the formation of autophagosomes by activating the expression of key autophagic molecules, such as LC3-II, beclin-1, and ATG7, in IPF fibroblasts and BLM-induced fibrotic lung tissues (Fig. 7). Spermidine also suppressed mTOR, a downstream mediator of PI3K/AKT signaling that inhibits macroautophagy^56^ and is part of an ATG7-dependent canonical pathway (Fig. 7d). These data confirm that spermidine activates autophagy in IPF fibroblasts and experimental lung fibrosis. One study demonstrated impaired autophagy in the lung tissues of patients with IPF, as indicated by p62 accumulation and decreased LC-II expression^33^. Decreased autophagy-mediated clearance of ECM proteins leads to their accumulation, contributing to the progression of fibrosis. Recently, Kim et al. reported that the induction of autophagy alleviates BLM-induced lung fibrosis^21^. Thus, spermidine-mediated enhancement of autophagy may help resolve fibrosis.

Spermidine is a ubiquitous polycation that is associated with several important cellular functions and helps to maintain general cell homeostasis. Polyamines are essential for cell growth and proliferation and tissue regeneration. Polyamines bind and stabilize DNA and RNA, have antioxidant activities, modulate enzyme functions and are required for the regulation of translation^57^. Spermidine acts as a natural autophagy inducer and antiaging compound and exhibits many beneficial effects, such as cardioprotective, neuroprotective, and antitumorigenic effects^14^. In polyamine metabolism, spermidine is formed from its precursor putrescin or by the degradation of spermine, and its biosynthesis is connected to arginine and nitric oxide metabolism via ornithine^58^. North et al. reported increased airway spermidine levels in asthma and an association with enhanced airway hyperresponsiveness through the inhibition of constitutive NO synthesis^59^. To the best of our knowledge, only two studies have examined the levels of spermidine in lung tissues with IPF using mass spectrometry. We previously found that spermidine levels were 30% lower in IPF lungs than in controls (Supplementary Fig. 3)^16^. However, Zhao et al. reported increased spermidine levels in IPF lungs^60^. We hypothesize that this discrepancy may be due to different sample numbers and to the different stages of IPF in lung tissue. This study is the first to report the effects of exogenous spermidine on experimental lung fibrosis and to investigate its possible mechanism.

In conclusion, exogenous spermidine administration attenuated lung inflammation and fibrosis in established BLM-induced experimental fibrosis. These findings were associated with decreased cellular senescence, ER stress-related apoptosis in AECs, and the induction of autophagy. Taken together, these data suggest that spermidine may be a promising therapeutic agent for fibrotic lung diseases, including IPF.

The English in this document has been checked by at least two professional editors, both of whom are native speakers of English. For a certificate, please see: http://www.textcheck.com/certificate/rFdygU.

## Supplementary information

Supplementary information
