# Supplementary material for: Spermidine attenuates bleomycin-induced lung fibrosis by inducing autophagy and inhibiting endoplasmic reticulum stress (ERS)-induced cell death in mice
Source: Exp Mol Med. 2020 Dec 14;52(12):2034–45. doi: 10.1038/s12276-020-00545-z (PMC8080799; doi:10.1038/s12276-020-00545-z)
Supplement: Supplementary file 1 — Supplementary information [file 12276_2020_545_MOESM1_ESM.docx]

**Spermidine attenuated bleomycin induced lung injury/fibrosis by inducing autophagy and inhibition of endoplasmic reticulum stress (ERS) induced cell death in mice**

Ae Rin Baek, M.D.* Jisu Hong. M.S. *, Ki Sung Song. M.S. ^*^, An Soo Jang, M.D., Ph.D.*, Do Jin Kim, M.D., Ph.D.*, Su Sie Chin, M.D., Ph.D.^†^, Sung Woo Park, M.D., Ph.D.*

*Division of Allergy and Respiratory Medicine, Department of Internal Medicine, Soonchunhyang University Bucheon Hospital, 14584, Gyeonggi-Do, south Korea. ^†^Department of Pathology, Soonchunhyang University Bucheon Hospital, 14584, Gyeonggi-Do, south Korea.

**Supplementary information**


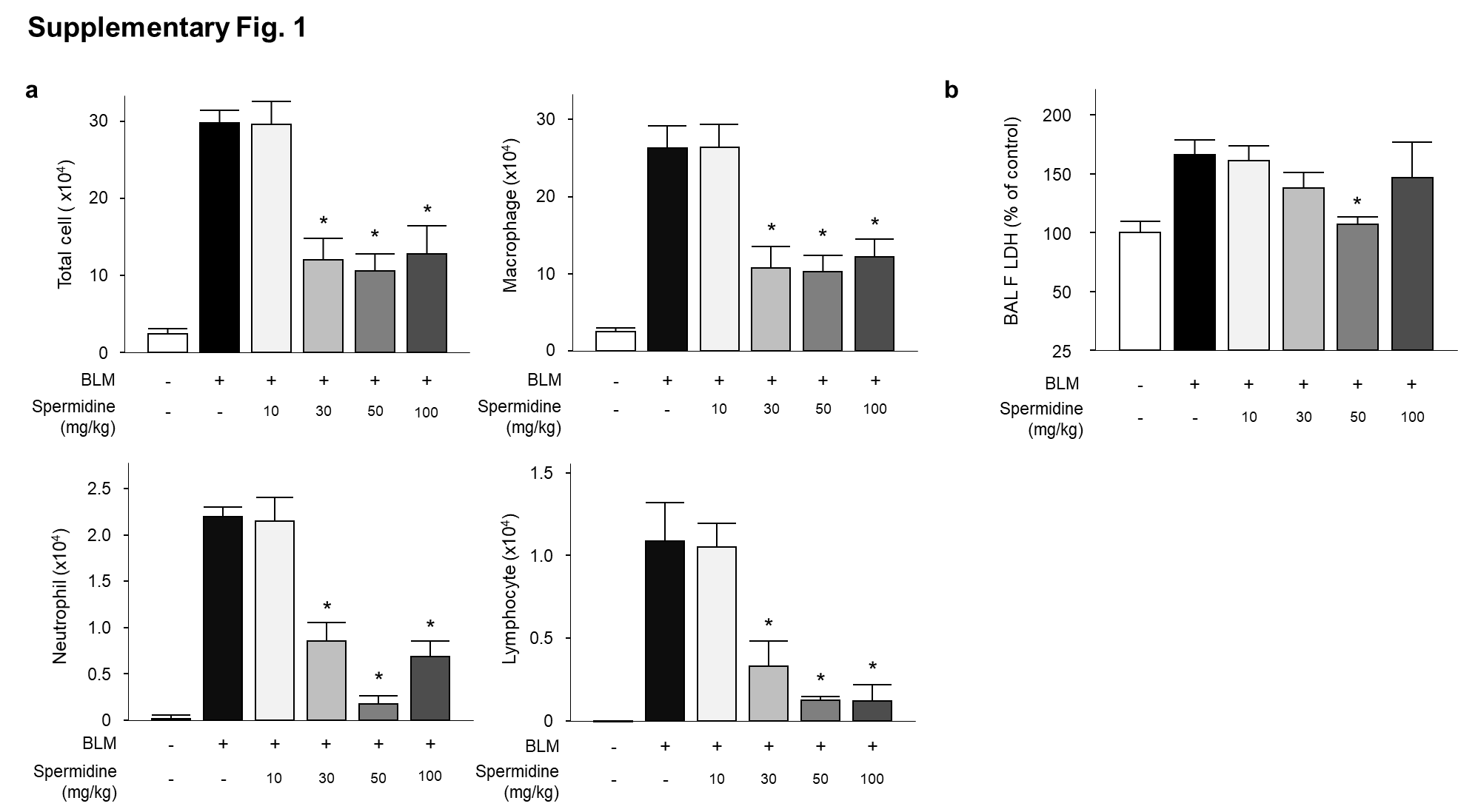


**Supplementary Figure 1. Measurement of BAL fluid cellular analysis and LDH levels**

BLM-treated mouse treated with various doses of spermidine intraperitoneally on days 10–21. BAL samples were collected on day 21**. a.** The total number of cells was counted using a hemocytometer. Differential cell counts in BAL fluid were analyzed from 500 cells stained with Diff-Quik (n = 4/group). **b.** BAL fluid LDH levels were measured by LDH assay kit (n = 4/group). **p* <0.05 vs. BLM(+)/spermidine(–)treated group.


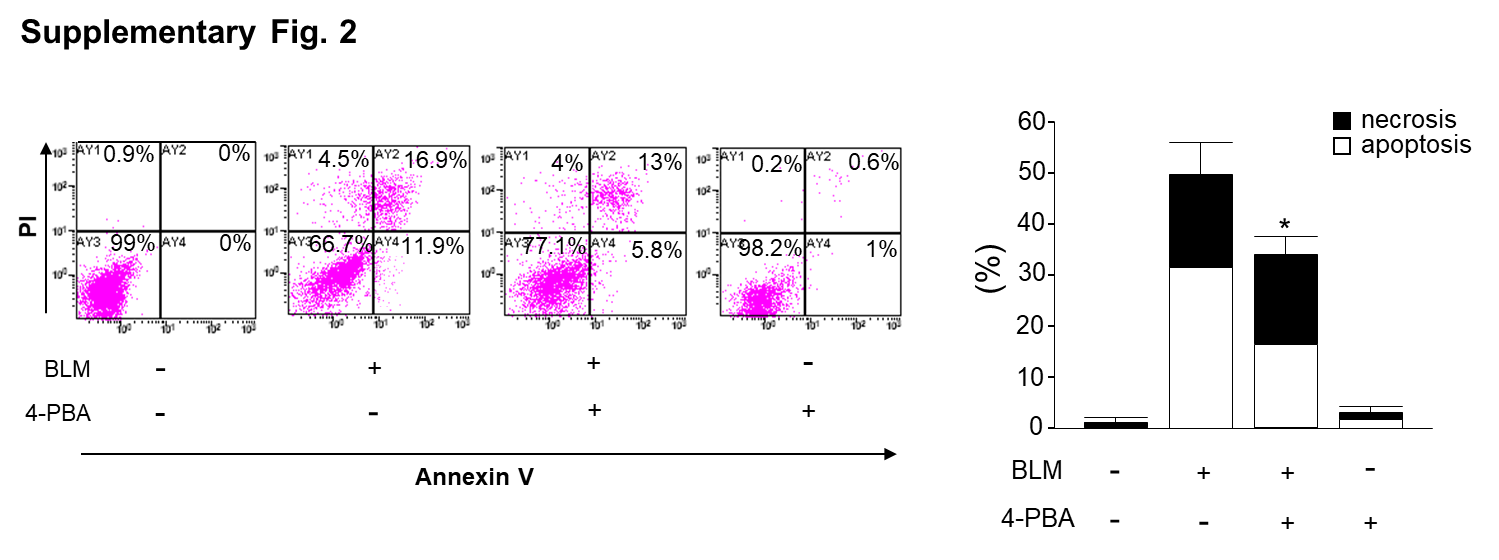


**Supplementary Figure 2.**

Primary mouse AECs were exposed to BLM (10 µg/mL) and/or 4-PBA (5 mM), an ER stress inhibitor, for 24 h in serum-free medium. **p* <0.05 vs. apoptosis of the BLM(+)/4-PBA (–) treated groups. Apoptosis was defined as annexin V(+)/both PI(+) and PI(–). Necrosis was defined as PI(+)/both annexin V(+) and annexin V(–). (n = 3)


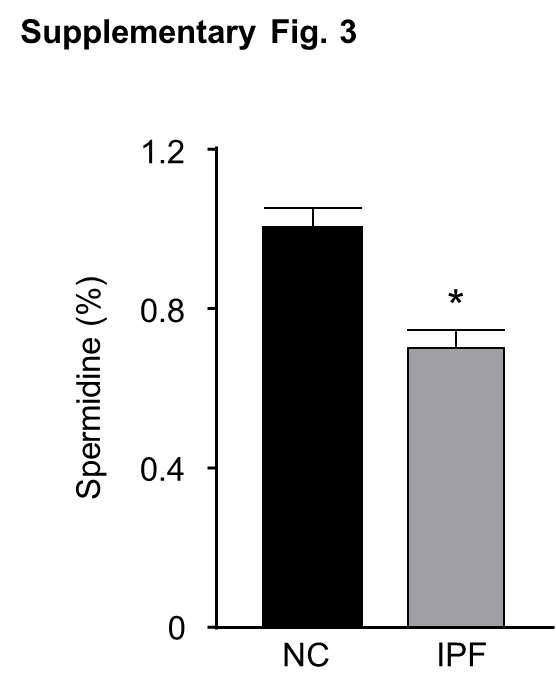


**Supplementary Figure 3.**

Relative abundance of spermidine. Data are shown as means and SEM.

**p* <0.05 vs. controls.
